# A Manual of Obstetrics

**Published:** 1900-12

**Authors:** 


A Manual of Obstetrics.—By A. F. A. King, M. D., Professor of Obstetrics and Diseases of Women in the Medical Department of the Columbian University, Washington, D. C., and in the University of Vermont, etc. In one 12mo. volume of 612 pages, with 264 illustrations. Cloth, $2.50, net. Lea Brothers & Co., publishers, Philadelphia and New York.

This book has gone through seven full editions, and is appreciated thoroughly by all classes of the profession. It is so clear and trustworthy that it takes the place with many physicians of a more extensive work. It is up-to-date and has forty-one new engravings added to its heretofore richly illustrated pages. T. J. B.

